# 595. The Effect of Herpes Zoster Vaccination on New Diagnoses of Dementia: A Quasi-randomized Study in Australia

**DOI:** 10.1093/ofid/ofae631.190

**Published:** 2025-01-29

**Authors:** Michael Pomirchy, Christian Bommer, Pascal Geldsetzer

**Affiliations:** Stanford University, Palo Alto, California; Stanford University, Palo Alto, California; Stanford University, Palo Alto, California

## Abstract

**Background:**

We have recently provided evidence from a quasi-randomized study in Wales that herpes zoster (HZ) vaccination reduced the incidence of new dementia diagnoses. This study used a similar quasi-randomization in Australia to determine the effect of HZ vaccination on new dementia diagnoses over 7.5 years.

**Figure 1:**
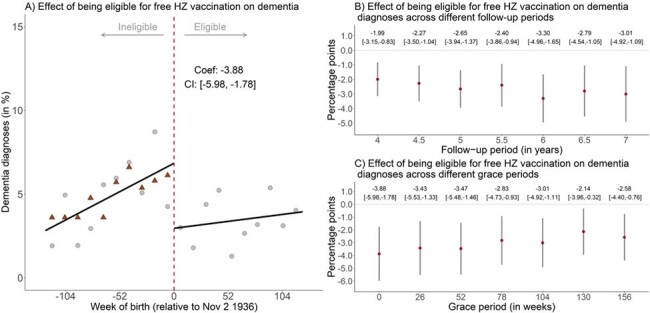
Eligibility for a free herpes zoster (HZ) vaccine reduces dementia diagnoses In panel (a), a regression discontinuity is plotted, where the forcing variable is the number of weeks from the cutoff (November 2, 1936) and the outcome is dementia diagnoses (in percentages). The triangular dots indicate dementia diagnoses for the comparison group, scaled up by the intercept for the main cohort. Panel (b) describes this effect for different follow-up periods, and panel (c) shows the effect for different grace periods.

**Methods:**

In Australia, starting on November 1 2016, live-attenuated HZ vaccination was provided for free to individuals aged 70 to 79 years of age. Thus, those whose 80^th^ birthday was just a few days prior to November 1 2016 never became eligible, whereas those whose 80^th^ birthday was a few days later were eligible for one year. There is no reason to expect that these population groups who differ in their age by only a minute degree should differ in any of their dementia-related characteristics. We used detailed primary healthcare records (provided by PenCS) with week-of-birth information from 65 general practices across Australia. We analyzed our data using a comparative regression discontinuity design, with dementia diagnoses from older birth cohorts serving as a comparison group.

**Results:**

Of 108,670 patients in our data, 15,297 were born between November 2 1928 and November 2 1944. At baseline, patients were well-balanced across the date-of-birth eligibility threshold (November 2 1936) for HZ vaccination in their preventive health services uptake and chronic disease diagnoses. There was an abrupt increase of 13.2 (95% CI: [8.7, 17.6], p = 0.001) percentage points in the probability of ever receiving HZ vaccination between patients born shortly before versus shortly after the eligibility threshold. Eligibility for a free HZ vaccination caused a 3.9 percentage point (95% CI: [1.7, 6.1], p = 0.001) decrease in the probability of receiving a new dementia diagnosis over 7.5 years. Being eligible for HZ vaccination did not affect the probability of taking up other preventive health services (including other vaccinations) nor do we find consistent evidence that it affected the probability of receiving any of the twenty most common disease diagnoses in our data.

**Conclusion:**

In conjunction with our findings from Wales, these results from Australia constitute robust evidence that live-attenuated HZ vaccination may delay or prevent dementia.

**Disclosures:**

**All Authors**: No reported disclosures

